# Comparative Study between Surgical Repair of Atrial Septal Defect via Median Sternotomy, Right Submammary Thoracotomy, and Right Vertical Infra-Axillary Thoracotomy

**DOI:** 10.21470/1678-9741-2019-0096

**Published:** 2020

**Authors:** Zeng-rong Luo, Qiang Chen, Ling-li Yu, Liang-wan Chen, Zhong-yao Huang

**Affiliations:** 1 Department of Cardiovascular Surgery, Union Hospital, Fujian Medical University, Fuzhou, Fujian, People’s Republic of China.

**Keywords:** Heart Septal Defects, Atrial, Cardiopulmonary Bypass, Sternotomy, Surgical Wound, Respiration, Artificial, Postoperative Complications

## Abstract

**Objective:**

To compare the results of surgical repair via median sternotomy, right submammary thoracotomy, and right vertical infra-axillary thoracotomy for atrial septal defect (ASD).

**Methods:**

This is a retrospective analysis of the relative perioperative and postoperative data of 136 patients who underwent surgical repair for ASD with the abovementioned three different treatments in our hospital from June 2014 to December 2017.

**Results:**

The results of the surgeries were all satisfactory in the three groups. No statistically significant difference was found in operative time, duration of cardiopulmonary bypass, blood transfusion amount, postoperative mechanical ventilation time, duration of intensive care unit, length of hospital stay, and hospital costs. However, the median sternotomy group had the longest incision. Meanwhile, there was no significant difference in postoperative complications.

**Conclusion:**

All three types of surgical incisions can be safely and effectively used to repair ASD. The treatments via right submammary thoracotomy and right vertical infra-axillary thoracotomy have advantages over the treatment via median sternotomy in cosmetic results and should be the recommended options.

**Table:** 

Abbreviations, acronyms & symbols	
ASD	= Atrial septal defect
CPB	= Cardiopulmonary bypass
ICU	= Intensive care unit
M/F	= Male/Female
TTE	= Transthoracic echocardiography

## INTRODUCTION

Atrial septal defect (ASD) is one of the most common congenital heart malformations^[1,2]^. Surgical repair via median sternotomy is still considered as the traditional and golden treatment for ASD^[3-5]^. The advantage of such approach is that it is easy to expose the surgical field and complete the operation. However, it is also associated with a long incision. In recent years, the method of transcatheter device closure of ASD has been widely used in many cardiac centers, and it has mostly replaced the surgical treatment^[6,7]^. But there are still some cases which are not suitable for such treatment and require surgical repair. More attention has been paid to the cosmetic effects of the cardiac surgical field, especially in female patients, which lead to the constant change of incision location. In recent years, right posterolateral thoracotomy, right vertical infra-axillary thoracotomy, right submammary thoracotomy, and lower mini-sternotomy were often used to achieve a better cosmetic result^[8-14]^. In this paper, we want to compare the results of three different incisions for surgical repair of ASD (via median sternotomy, right submammary thoracotomy, and right vertical infra-axillary thoracotomy).

## METHODS

We retrospective collected and analyzed the medical records of 136 patients who underwent surgical repair of ASD in our hospital between June 2014 and December 2017. According to the different incision selected, the patients were divided into three groups: 45 patients (age, 3.38±1.00 years; gender male/female (M/F), 28/17; and weight, 12.51±1.81 kg) in group A, who underwent median sternotomy; 42 patients (age, 3.15±0.81 years; gender M/F, 25/17; and weight, 12.53±1.69 kg) in group B, who underwent right submammary thoracotomy; and 49 patients (age, 3.25±0.25 years; gender M/F, 29/20; and weight, 12.36±1.56 kg) in group C, who underwent right vertical infra-axillary thoracotomy. Of all the patients, eight cases were converted to surgical repair because of failing device occlusion. The inclusion criteria were isolated ASD with/without mild-moderate pulmonary hypertension and the fact that most of the patients were not suitable for transcatheter device closure. The exclusion criteria were ASD concomitant with other intracardial malformations, severe pulmonary hypertension or Eisenmenger syndrome, ASD combined with other organ diseases, and consent not obtained from the patient or their relatives. Routine preoperative clinical examinations were performed, which included an electrocardiogram, a chest X-ray film, and routine blood and biochemical tests. The confirmed diagnosis of ASD was performed by transthoracic echocardiography (TTE). From Table 1, we can conclude that there were no significant differences in age, gender, body weight distribution, and the diameter of ASD among these three groups.

**Table 1:** Comparison of preoperative data among three groups of patients.

Item	Group A	Group B	Group C	*P*-value
N	45	42	49	
Age (years)	3.38±1.00	3.15±0.81	3.25±0.25	0.326
Gender (M/F)	28/17	25/17	29/20	0.923
Weight (kg)	12.51±1.81	12.53±1.69	12.36±1.56	0.841
Size of ASD (mm)	14.76±2.32	14.45±2.23	14.17±2.12	0.792
Pulmonary hypertension (mmHg)	33.00±3.35	31.43±2.99	32.25±3.01	0.574
Cardiothoracic ratio	0.52±0.15	0.53±0.13	0.52±0.14	0.784

This study was approved by the ethics committee of Fujian Medical University, China and adhered to the tenets of the Declaration of Helsinki. Additionally, written informed consent was obtained from the patients’ relatives.

### Surgical Repair Technique Via Median Sternotomy in Group A

**Fig. 1:**
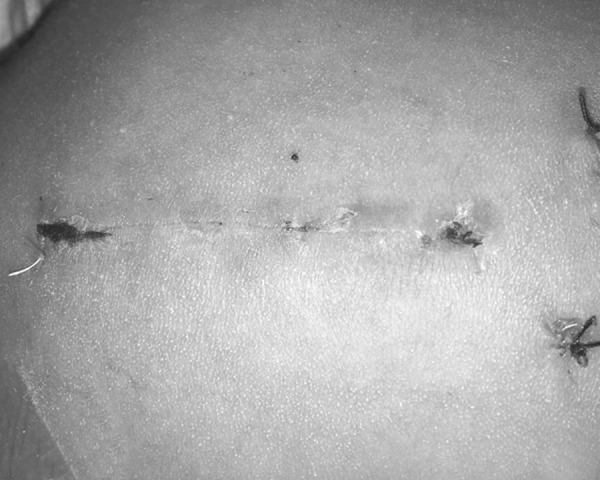
The median incision in group A.

This treatment was conducted through a median sternotomy approach, under cardiopulmonary bypass (CPB), without ascending aorta cross-clamping, and the sternum was split entirely. The pericardial patch was used as repair material in all patients (Figure 1).

### Surgical Repair Technique Via Right Submammary Thoracotomy in Group B

**Fig. 2:**
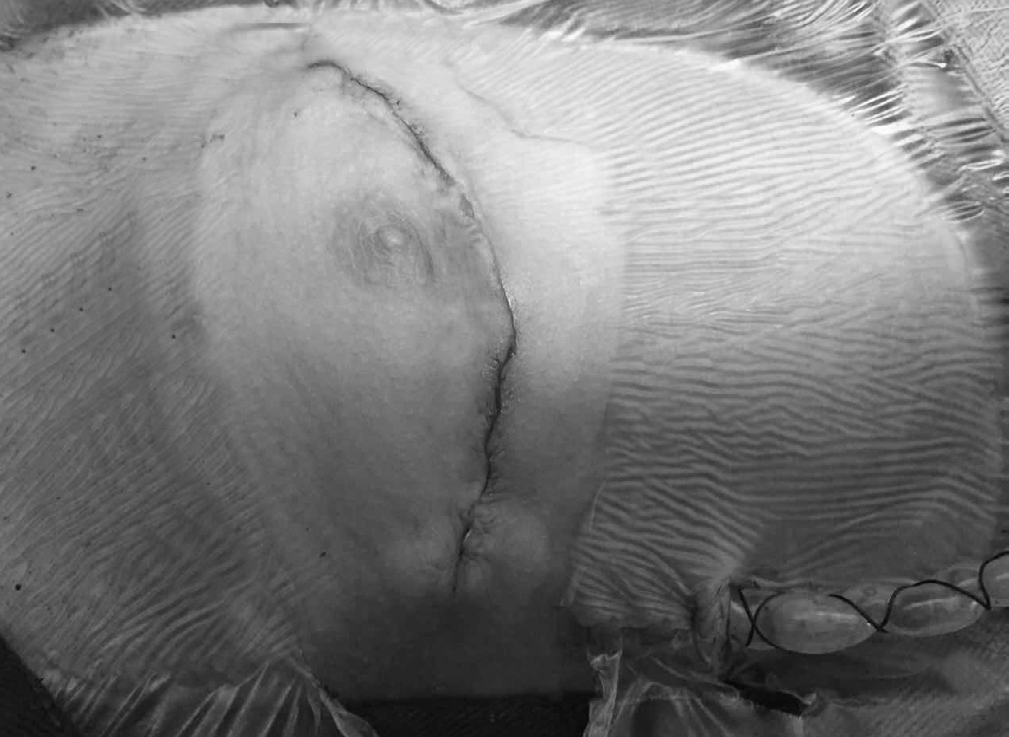
The right submammary incision in group B.

The patient was placed in a recumbent position with a cushion tilted at 30 to 40 degrees on the right chest. We chose the curved incision at the lower margin of the right submammary groove as the surgical approach (Figure 2), then we made the incision through the fourth or fifth intercostal space. A small rib spreader was used to open the intercostal space, and the pericardium was opened to expose the surgical field (including the ascending aorta root, right atrium, partial of the right ventricle, and the upper and inferior vena cava). After systemic heparinization, routine cannulations were performed to establish a standard CPB. Likewise, no aortic cross-clamp and cardiac arrest were required. Other surgical procedures for ASD closure were the same as used in the group A. Finally, we closed the incision with a continuous intradermal suture for the skin.

### Surgical Repair Technique Via Right Vertical Infra-Axillary Thoracotomy in Group C

**Fig. 3:**
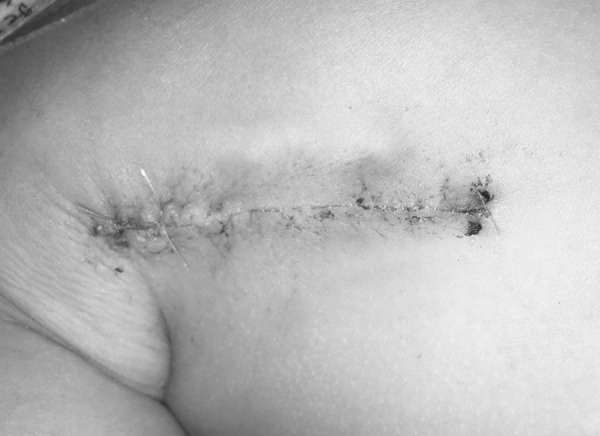
The right vertical infra-axillary incision in group C.

The patient was placed in a left lateral decubitus position with the right arm being held at right angles to the head and the shoulder joint being swung back as far as possible. The incision was selected in the middle line of the right axilla, from the second intercostal to the fifth intercostal space, and the length was about 5-7 cm, being adjusted according to the patient's weight and height (Figure 3). The right thoracic cavity was opened through the third or fourth intercostal space, and a wet sponge was used to obstruct the right lung to expose the pericardium. The pericardium was cut and suspended, exposing the aortic root and the superior and inferior vena cava. After heparinization, the routine establishment of CPB and ASD closure were the same as for the previous groups A and B.

### Data Collection

The clinical perioperative data (including operative time, duration of CPB, blood transfusion amount, the incision length, postoperative mechanical ventilation time, duration of intensive care unit (ICU), length of hospital stay, and hospital cost) (Table 2) and postoperative complications (Table 3) of all patients were collected and analyzed. During the 3-month follow-up, all patients were routinely conducted physical examination, electrocardiogram, and TTE.

**Table 2:** Comparison of perioperative and postoperative data among three groups of patients.

Item	Group A	Group B	Group C	*P*-value
Operative time (min)	85.81±5.67	90.34±7.43	92.95±6.54	0.532
Cardiopulmonary bypass time (min)	34.37±4.58	36.65±5.31	37.98±6.12	0.813
Mechanical ventilation time (h)	5.92±1.37	5.42±1.21	5.67±1.18	0.350
Intensive care unit time (h)	14.96±2.17	13.86±1.91	14.31±1.85	0.316
Drainage (ml)	81.53±13.18	62.48±9.51*	65.19±9.75*	0.000
Blood transfusion volume (ml)	301.82±49.49	286.29±40.65	274.56±36.72	0.184
The incision length (cm)	8.07±1.27	6.45±0.76*	6.04±0.84*	0.020
Postoperative hospital stay (days)	4.67±0.96	4.54±0.87	4.35±0.68	0.904
Hospital costs (10000RMB**)	3.02±0.32	2.95±0.29	2.97±0.26	0.568

* *P*<0.05 compared with group A

** Costs in renminbi (the Chinese currency)

**Table 3:** Comparison of postoperative complications among three groups of patients.

Item	Group A	Group B	Group C	*P*-value
Cerebrovascular accident	0	0	0	
Large residual shunt	0	0	0	
Severe arrhythmia	0	0	0	
Low cardiac output syndrome	0	0	0	
Pulmonary infection	2	3	4	0.795
Pneumothorax	0	1	1	0.378
Subcutaneous emphysema	0	1	1	0.378
Thoracic deformity	4	0	0	0.010
Pericardial effusion	1	0	0	0.323
Pleural effusion	0	2	2	0.403

### Statistical Analysis

Continuous variables were expressed as x±s. Analysis of variances was applied for continuous variables and the χ^2^ or Fisher's test for categorical variables. A *P*-value <0.05 was defined as statistical significance.

## RESULTS

All the patients underwent successful closure without fatal complications, such as postoperative low cardiac output syndrome, death, malignant arrhythmia, reoperation for ASD, multiple organ dysfunction, or cerebrovascular accident. There were no statistically significant differences in operative time, duration of CPB, blood transfusion amount, postoperative mechanical ventilation time, duration of ICU, length of hospital stay, and hospital cost among these three groups.

However, pericardial effusion only occurred in group A, while pleural effusion occurred in the other two groups. The group A had the longest incision while the other two groups had relatively smaller incisions. The incidence of postoperative pulmonary infection was similar among these three groups. Moreover, the incidence of postoperative pneumothorax and subcutaneous emphysema was slightly higher in groups B and C than in group A, which was not a statistically significant difference.

During the follow-up period, there were no severe complications in all patients. Malunion of the sternum resulting from surgery occurred in four cases in group A, including three cases with pectus carinatum and one case with funnel chest. These patients were asymptomatic and complained about cosmetic issues during the follow-up period. No further surgical intervention was needed in these patients, except for close medical observation. There was no chest deformity in the other two groups.

## DISCUSSION

Surgical repair with a median incision for most of the simple congenital heart diseases has been proved with a very good clinical effect^[3-5]^. However, the surgical scar was associated with cosmetic issues and adverse psychological factor, which affected the patient’s quality of life^[15,16]^. In recent years, the transcatheter interventional procedure has been developed rapidly because of its better cosmetic effect^[6,7]^. But at the same time, it is often reported that the acute and delayed complications caused by transcatheter interventional procedure need to be treated, and the complications of such emergency operation are significantly higher than those of elective surgery^[17]^. Based on the analysis and research of the mid- and long-term follow-ups of the transcatheter interventional procedure, some warning reports about the peripheral vascular injury, the occluder dislodgement, and the late-onset complete atrioventricular block have been received more and more attention^[18]^. Although there have been some reports about robotic and thoracoscopic-assisted closure of ASD, the disadvantages of these procedures were high technical requirements, high cost, and being challenging to popularize^[19-21]^. So, traditional surgical techniques are still desirable. Accordingly, various minimally invasive and cosmetic surgical incisions have been adopted, which includes the lower mini-sternotomy incision, right submammary incision, right vertical infra-axillary incision, and right posterolateral thoracotomy incision^[8-14]^.

In our cardiac center, we had repaired ASD through the median sternotomy, the right submammary thoracotomy, and the right vertical infra-axillary thoracotomy. In a literature review, there were few reports about the comparison of these three surgical incisions^[22-24]^. In the comparison of these three groups, there were no significant differences in operative time, duration of CPB, and blood transfusion amount, which means that different incisions did not make an operation more difficult. Although the operative techniques and intubation in groups B and C were more difficult to execute than those in group A, skilled surgeons can overcome these problems. The late rapid chest closing technique in groups B and C had time-saving advantages, which made the whole operative time similar to group A. None of the patients underwent ascending aorta cross-clamping, which may dramatically shorten the time of CPB and facilitate the postoperative recovery. The corresponding postoperative mechanical ventilation time, duration of ICU, and length of hospital stay were almost identical. Different from the robotic and thoracoscopic-assisted surgery^[19-21]^, there were not any other additional surgical instruments in the groups B and C, so the hospital cost was almost similar among these three groups, which also made these techniques accessible to developers in the general cardiac center.

Regarding complications, none of the three groups had severe or fatal events, which also confirmed the safety of these procedures. In group A, pericardial effusion may be associated with a split sternum, but such complication had not led to severe consequences. Although the intraoperative pleural cavity was exposed and the lung tissue was compressed, it did not increase the risk of postoperative pulmonary infection in groups B and C. Postoperative pneumothorax and subcutaneous emphysema occurred in a small number of patients in groups B and C, which were easy to treat by replacement of the drainage tube. The thoracic deformity only occurred in group A, and there were no ways to avoid it. To overcome this drawback, the choice of the other two incisions seemed to be a better approach. However, regarding the median incision, its relatively short opening of the chest and best surgical field exposure are ideal for those emergency surgeries where there is an occluder dislodgement.

For surgeons, there are many options for the incision and the risks and outcomes of the different treatments are independent of the surgical approach. The different approaches have their limitations for the exposure of the heart and intracardial structures and may lead to damage to adjacent structures. For groups B and C, there are some technical points. In group B, the method seemed to present a risk of damage to the breast tissue and the muscle of the chest. Therefore, it is not suitable for young female and adult patients. When it is applied to minors, the incision should be made away from the breast tissue so that they do not affect breast development^[25-28]^. For group C, the method was not suitable for older children due to the limitation of chest depth. So, individualized selection is also necessary, and the surgeons' proficiency level should also be considered. The arterial cannula is another key to successful surgery. Because of the higher position of the ascending aorta and the right auricle covering the ascending aorta, it is necessary to suspend the right auricle to expose the aortic root satisfactorily and create good conditions for the smooth cannula. In group C, pericardium suspension should be done to expose the cannula site due to the deep thorax^[29,30]^. Arterial cannula must be performed with the assistance of vascular forceps to ensure accurate cannula. It should be carefully done to not to squeeze the lungs during surgery in these two groups and to fully expand the lungs after surgery, which can prevent postoperative atelectasis and pulmonary infection. Besides, it should be taken care to prevent damage to the posterior wall of the right atrium during the venous cannula, where bleeding is difficult to repair. So, the surgeon must be aware of the potential risks of different approaches, such as the incisions in groups B and C, of which exposure was significantly better in young children than in adults, and of the fact that the aortic and vena cava intubation was more difficult with age and weight gain.

This study has the following limitations: first, this is a retrospective study, not a randomized controlled prospective study and there is some selection bias in the patients’ groups, but the results still have some clinical significance. Secondly, the results were conducted in a single center, and the sample size is small, so other cardiac units may have different results. Also, long-term follow-up results need to be further summarized.

## CONCLUSION

Surgical repair of ASD via median sternotomy, right submammary thoracotomy, and right vertical infra-axillary thoracotomy can present satisfactory clinical results with few complications, but the latter two incisions have an excellent cosmetic result. Under the premise that the operation can be completed safely, skilled cardiac surgeons should choose the different incisions according to the patients' age, sex, weight, and cosmetic demand.

**Table:** 

Author's roles & responsibilities	
ZRL	The acquisition, analysis, or interpretation of data for the work; final approval of the version to be published
QC	Substantial contributions to the conception or design of the work; or the acquisition, analysis, or interpretation of data for the work; drafting the work or revising it critically for important intellectual content; final approval of the version to be published
LLY	The acquisition, analysis, or interpretation of data for the work; final approval of the version to be published
LWC	Final approval of the version to be published
ZYH	Substantial contributions to the conception or design of the work; or the acquisition, analysis, or interpretation of data for the work; drafting the work or revising it critically for important intellectual content; final approval of the version to be published

## References

[1] Vasquez AF, Lasala JM (2013). Atrial septal defect closure. Cardiol Clin.

[2] Geva T, Martins JD, Wald RM (2014). Atrial septal defects. Lancet.

[3] Moake L, Ramaciotti C (2005). Atrial septal defect treatment options. AACN Clin Issues.

[4] Murphy JG, Gersh BJ, McGoon MD, Mair DD, Porter CJ, Ilstrup DM (1990). Long-term outcome after surgical repair of isolated atrial septal defect. Follow-up at 27 to 32 years. N Engl J Med.

[5] Horvath KA, Burke RP, Collins JJ Jr, Cohn LH (1992). Surgical treatment of adult atrial septal defect: early and long-term results. J Am Coll Cardiol.

[6] Aytemir K, Oto A, Özkutlu S, Canpolat U, Kaya EB, Yorgun H (2013). Transcatheter interatrial septal defect closure in a large cohort: midterm follow-up results. Congenit Heart Dis.

[7] Law MA, Josey J, Justino H, Mullins CE, Ing FF, Nugent AW (2009). Long-term follow-up of the STARFlex device for closure of secundum atrial septal defect. Catheter Cardiovasc Interv.

[8] De Mulder W, Vanermen H (2002). Repair of atrial septal defects via limited right anterolateral thoracotomy. Acta Chir Belg.

[9] Panos A, Aubert S, Champsaur G, Ninet J (2003). Repair of atrial septal defect through a limited right anterolateral thoracotomy in 242 patients: a cosmetic approach?. Heart Surg Forum.

[10] Bozso SJ, Grant A, Iglesias I, Chu MWA (2016). Minimally invasive periareolar approach to unroofed coronary sinus atrial septal defect repair. Ann Thorac Surg.

[11] Vida VL, Tessari C, Fabozzo A, Padalino MA, Barzon E, Zucchetta F (2013). The evolution of the right anterolateral thoracotomy technique for correction of atrial septal defects: cosmetic and functional results in prepubescent patients. Ann Thorac Surg.

[12] Dave HH, Comber M, Solinger T, Bettex D, Dodge-Khatami A, Prêtre R (2009). Mid-term results of right axillary incision for the repair of a wide range of congenital cardiac defects. Eur J Cardiothorac Surg.

[13] Kaneda T, Nishino T, Saga T, Nakamoto S, Ogawa T, Satsu T (2013). Small right vertical infra-axillary incision for minimally invasive port-access cardiac surgery: a moving window method. Interact Cardiovasc Thorac Surg.

[14] Giamberti A, Mazzera E, Di Chiara L, Ferretti E, Pasquini L, Di Donato RM (2000). Right submammary minithoracotomy for repair of congenital heart defects. Eur J Cardiothorac Surg.

[15] Crossland DS, Jackson SP, Lyall R, Hamilton JR, Hasan A, Burn J (2005). Patient attitudes to sternotomy and thoracotomy scars. Thorac Cardiovasc Surg.

[16] Kantoch MJ, Eustace J, Collins-Nakai RL, Taylor DA, Bolsvert JA, Lysak PS (2006). The significance of cardiac surgery scars in adult patients with congenital heart disease. Kardiol Pol.

[17] Mellert F, Preusse CJ, Haushofer M, Winkler K, Nill C, Pfeiffer D (2001). Surgical management of complications caused by transcatheter ASD closure. Thorac Cardiovasc Surg.

[18] Chessa M, Carminati M, Butera G, Bini RM, Drago M, Rosti L (2002). Early and late complications associated with transcatheter occlusion of secundum atrial septal defect. J Am Coll Cardiol.

[19] Xiao C, Gao C, Yang M, Wang G, Wu Y, Wang J (2014). Totally robotic atrial septal defect closure: 7-year single-institution experience and follow-up. Interact Cardiovasc Thorac Surg.

[20] Zhe Z, Kun H, Xuezeng X, Yunge C, Zengshan M, Huiming G (2014). Totally thoracoscopic versus open surgery for closure of atrial septal defect: propensity-score matched comparison. Heart Surg Forum.

[21] Xu M, Zhu S, Wang X, Huang H, Zhao J (2015). Two different minimally invasive techniques for female patients with atrial septal defects: totally thoracoscopic technique and right anterolateral thoracotomy technique. Ann Thorac Cardiovasc Surg.

[22] Basaran M, Kocailik A, Ozbek C, Ucak A, Kafali E, Us M (2008). Comparison of 3 different incisions used for atrial-septal defect closure. Heart Surg Forum.

[23] Luo H, Wang J, Qiao C, Zhang X, Zhang W, Song L (2014). Evaluation of different minimally invasive techniques in the surgical treatment of atrial septal defect. J Thorac Cardiovasc Surg.

[24] Yaliniz H, Topcuoglu MS, Gocen U, Atalay A, Keklik V, Basturk Y (2015). Comparison between minimal right vertical infra-axillary thoracotomy and standard median sternotomy for repair of atrial septal defects. Asian J Surg.

[25] Däbritz S, Sachweh J, Walter M, Messmer BJ (1999). Closure of atrial septal defects via limited right anterolateral thoracotomy as a minimal invasive approach in female patients. Eur J Cardiothorac Surg.

[26] Isik O, Ayik MF, Akyuz M, Daylan A, Atay Y (2015). Right anterolateral thoracotomy in the repair of atrial septal defect: effect on breast development. J Card Surg.

[27] Yoshimura N, Yamaguchi M, Oshima Y, Oka S, Ootaki Y, Yoshida M (2001). Repair of atrial septal defect through a right posterolateral thoracotomy: a cosmetic approach for female patients. Ann Thorac Surg.

[28] Bleiziffer S, Schreiber C, Burgkart R, Regenfelder F, Kostolny M, Libera P (2004). The influence of right anterolateral thoracotomy in prepubescent female patients on late breast development and on the incidence of scoliosis. J Thorac Cardiovasc Surg.

[29] Silva Lda F, Silva JP, Turquetto AL, Franchi SM, Cascudo CM, Castro RM (2014). Horizontal right axillary minithoracotomy: aesthetic and effective option for atrial and ventricular septal defect repair in infants and toddlers. Rev Bras Cir Cardiovasc.

[30] Prêtre R, Kadner A, Dave H, Dodge-Khatami A, Bettex D, Berger F (2005). Right axillary incision: a cosmetically superior approach to repair a wide range of congenital cardiac defects. J Thorac Cardiovasc Surg.

